# A Gilbert syndrome-associated haplotype protects against fatty liver disease in humanized transgenic mice

**DOI:** 10.1038/s41598-020-65481-4

**Published:** 2020-05-26

**Authors:** Steffen Landerer, Sandra Kalthoff, Stefan Paulusch, Christian P. Strassburg

**Affiliations:** Department of Internal Medicine I, University Hospital Bonn, 53127 Bonn, Germany

**Keywords:** Metabolic diseases, Risk factors, Non-alcoholic fatty liver disease

## Abstract

UDP-glucuronosyltransferases 1 A (UGT1A) enzymes are capable of detoxifying a broad range of endo- and xenobiotic compounds, which contributes to antioxidative effects, modulation of inflammation and cytoprotection. In the presence of low-function genetic *UGT1A* variants fibrosis development is increased in various diseases. This study aimed to examine the role of common *UGT1A* polymorphisms in NASH. Therefore, *htgUGT1A*-WT mice and *htgUGT1A*-SNP mice (carrying a common human haplotype present in 10% of the white population) were fed a high-fat Paigen diet for 24 weeks. Serum aminotransferase activities, hepatic triglycerides, fibrosis development and *UGT1A* expression were assessed. Microscopic examination revealed higher hepatic fat deposition and a significant induction of *UGT1A* gene expression in *htgUGT1A*-WT mice. In agreement with these observations, lower serum aminotransferase activities and lower expression levels of fibrosis-related genes were measured in *htgUGT1A*-SNP mice. This was accompanied by reduced PPARα protein levels in *htgUGT1A*-WT but not in SNP mice. Our data demonstrate a protective effect of a *UGT1A* SNP haplotype, leading to milder hepatic steatosis and NASH. Higher PPARα protein levels in animals with impaired UGT1A activity are the likely result of reduced glucuronidation of ligands involved in PPARα-mediated fatty acid oxidation and may lead to the observed protection in *htgUGT1A*-SNP mice.

## Introduction

The growing number of patients with non-alcoholic fatty liver disease (NAFLD) renders this condition one of the most important and common liver disorder worldwide and thus to a significant medical challenge. It is estimated that around 30% of the general American population is affected by NAFLD and more than 85% of these individuals are morbidly obese^1,2^. The prevalence for NAFLD is closely related to obesity and its associated comorbidities and thus leads to an increased mortality rate^3,4^. In view of the high numbers of obese individuals, it is likely that the global incidence of patients with NAFLD will continue to increase. Apart from dietary habits further influences such as gender, age, ethnicity and genetic disorders have been identified as additional risk factors for NAFLD^5^, which has the potential to progress to more severe non-alcoholic steatohepatitis (NASH), cirrhosis and hepatocellular carcinoma (HCC)^6,7^. The molecular events include lobular inflammation, oxidative stress, hepatocellular apoptosis and fibrosis leading to NASH in approximately one quarter of NAFLD individuals^8,9^.

The development of a variety of liver diseases is almost invariably associated with a deregulation of nuclear receptor activation and changes in hepatic enzyme expression patterns. Previous studies have shown that the presence of NAFLD modulates the transcriptional activity of phase I and II enzymes^10,11^. Altered expression levels of hepatic UDP-glucuronosyltransferase *1a* (*Ugt1a*) genes have been detected in (*ob/ob*) mice^12^. Moreover, *Hardwick* and colleagues identified variations of *UGT1A* expression levels in human liver samples during various stages of NAFLD, but their role in NAFLD progression remain unclear^13^. UGT1A enzymes are localized in the inner membrane of the endoplasmatic reticulum and contribute to cytoprotection by catalysing the detoxification of a broad array of endo- and exogenous compounds. These include therapeutic drugs, environmental xenobiotics, reactive metabolites, bilirubin, bile acids, dietary fatty acids and other eicosanoids^14–16^. UGT1A proteins catalyse the covalent conjugation with glucuronic acid rendering lipophilic target substrates water soluble and inactive thereby facilitating biliary or renal elimination^17^. The presence of single nucleotide polymorphisms (SNPs) in the promoter and coding regions modifies the function of *UGT1A* genes^18^. Among more than 100 identified SNPs, which lead to varying degrees of UGT1A function and expression, the Gilbert syndrome-associated UGT1A1*28 variant probably represents the best studied *UGT1A* polymorphism^19^. Individuals homozygous for UGT1A1*28 exhibit a ~70% lower *UGT1A1* promoter activity^20^. Genetic *UGT1A* variants, commonly present in individuals with Gilbert syndrome, have been associated with several liver diseases including HCC and a more severe fibrosis development in patients with hepatitis B and C^21,22^. Based on these findings we designed experiments expecting that enhanced *UGT1A* expression confers a protective effect during hepatic steatosis, NASH development and, as a consequence, in the progression to liver fibrosis. Therefore, the aim of the study was to elucidate the role of *UGT1A* polymorphisms for NASH progression and determine the histopathological consequences for the liver. To this end, humanized transgenic (*htg*) *UGT1A* wild type (WT) and *htgUGT1A*-SNP mice, containing a human haplotype of 10 common occurring *UGT1A* SNPs, were used. Since this SNP haplotype is present in approx. 10% of the white population, our study further allows a risk assessment of NASH progression for a large proportion of the human population. Moreover, special interest was given to the nuclear receptor biology of farnesoid X receptor (FXR) and its downstream target peroxisome proliferator-activated receptor alpha (PPARα), which was shown to be downregulated in patients with fatty livers^23^. Both nuclear receptors have been identified as promising therapeutic targets for the treatment of NAFLD due to their ability to control a broad range of hepatic functions involved in lipid and glucose metabolism, inflammation and fibrogenesis^24,25^. Therefore, potential molecular mechanisms leading to the deregulation of FXR and PPARα activation possibly arising as a consequence of altered UGT1A activity in *htgUGT1A*-SNP mice are discussed and compared to the results of human population studies observed during NAFLD and NASH.

## Results

### Aggravated liver injury and inflammation in *htgUGT1A*-WT mice

**Figure 1:**
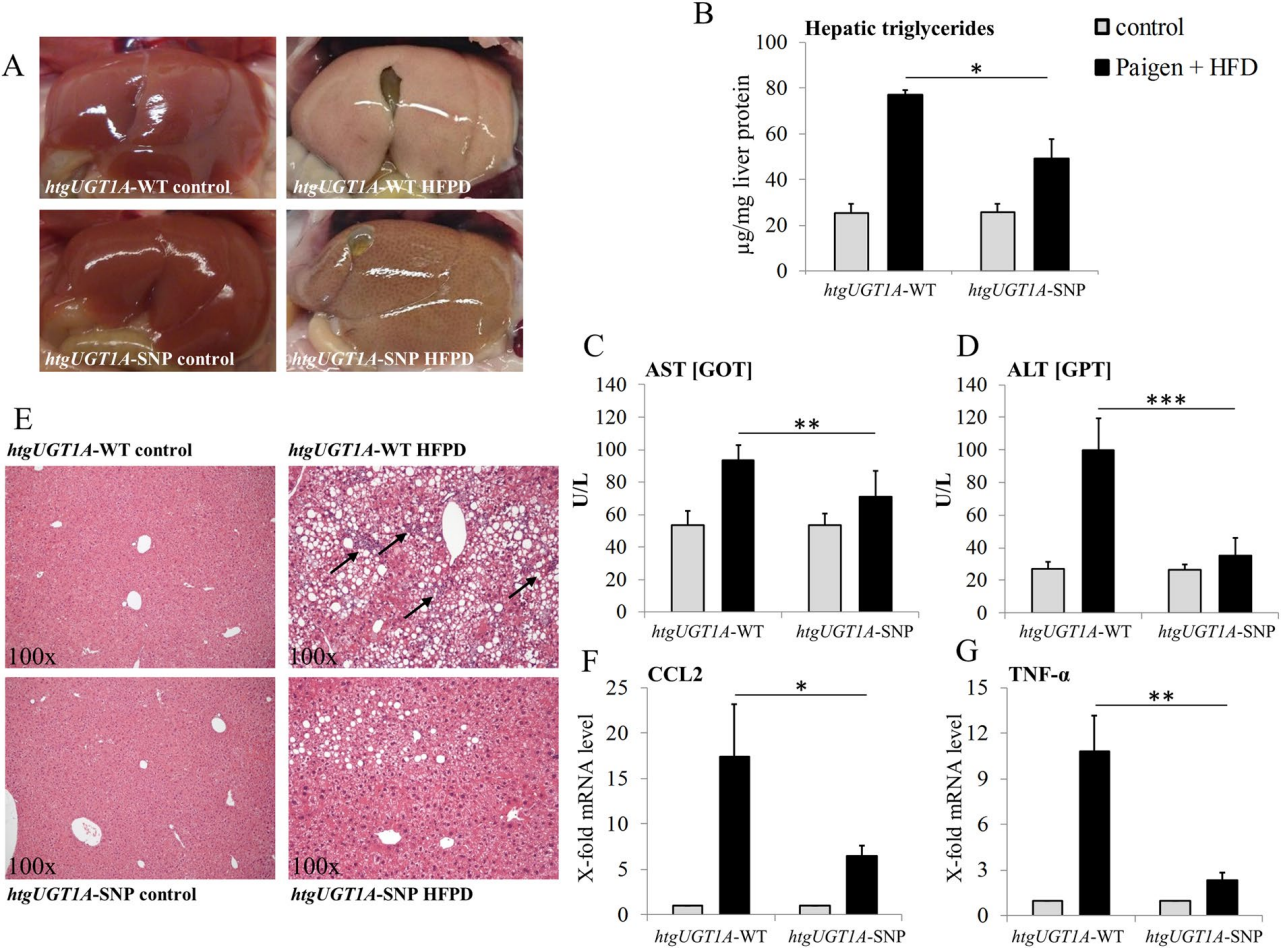
Differential effects of 24 weeks high-fat Paigen diet (HFPD) in *htgUGT1A*-WT and SNP mice. (**A**) Macroscopic liver depictions after abdominal opening. Livers of *htgUGT1A*-WT mice fed with HFPD showed a higher degree of steatosis as those of their SNP counterparts. (**B**) Hepatic triglyceride content expressed as µg/mg liver protein. In the presence of SNPs significantly lower levels of triglycerides were detected. (**C**) Representative sections of hematoxylin and eosin (H&E) stained liver tissue (magnification 100×). Hepatocytes of *htgUGT1A*-WT mice showed more fat accumulation and a higher proportion of infiltrated inflammation cells (black arrows). (**D,E**) Liver injury was assessed by serum aspartate aminotransferase (AST) and alanine aminotransferase (ALT) activities. Mice carrying the *UGT1A* SNP haplotype had significantly lower AST and ALT levels. (**F,G**) Gene expression levels of the pro-inflammatory markers C-C chemokine ligand 2 (CCL2) and tumour necrosis factor alpha (TNF-α). Induction of the transcriptional activation was significantly lower in *htgUGT1A*-SNP mice indicating a higher degree of inflammation. Each column represents the mean ± standard deviation. *p < 0.05; **p < 0.01; ***p < 0.001.

To elucidate the role of UGT1A enzymes in diet-induced liver injury, *htgUGT1A*-WT and SNP mice were fed with a high-fat Paigen diet (HFPD) for 24 weeks and analysed for differences regarding hepatic lipid deposition, triglyceride levels and serum aminotransferase activities. Contrary to our initial hypothesis, the macroscopic examination of the livers revealed higher fat deposition in *htgUGT1A*-WT mice (Fig. 1A). Measurement of hepatic triglycerides (Fig. 1B) confirmed the visual impression and revealed significantly higher triglyceride levels in *htgUGT1A*-WT animals (WT: 77.1 µg/mg; SNP: 49.0 µg/mg). In comparison to control diet fed animals, both mouse lines showed marked elevations of aspartate aminotransferase (AST) and alanine aminotransferase (ALT) activities after HFPD exposition (Fig. 1C,D). Interestingly, significantly lower AST (24%) and ALT (65%) levels were detected in in mice carrying the *UGT1A* SNP variant.

Hepatic steatosis was further analysed by haematoxylin-eosin (H&E) histological staining (Fig. 1E). In agreement with the differential hepatic lipid incorporation, HFPD-treated *htgUGT1A*-WT mice not only showed a higher proportion of steatotic hepatocytes, but also cellular ballooning that was almost exclusively observed in mice carrying the human wild type *UGT1A* gene locus. Moreover, an advanced degree of liver inflammation, indicated by the massive infiltration of inflammatory cells, was observed in *htgUGT1A*-WT mice suggesting the development of NASH (Fig. 1E black arrows). This observation was supported by significant differences in transcriptional activation of the proinflammatory marker genes C-C chemokine ligand 2 (CCL2) and tumour necrosis factor alpha (TNF-α) (Fig. 1F,G). In *htgUGT1A*-WT mice, a 17.4-fold CCL2 upregulation and a 10.8-fold TNF-α mRNA induction was detected, whereas in the presence of SNPs transcriptional activation of CCL2 (6.4-fold) and TNF-α (2.4-fold) was significantly less evident.

### Attenuated hepatic fibrosis in *htgUGT1A*-SNP mice

**Figure 2:**
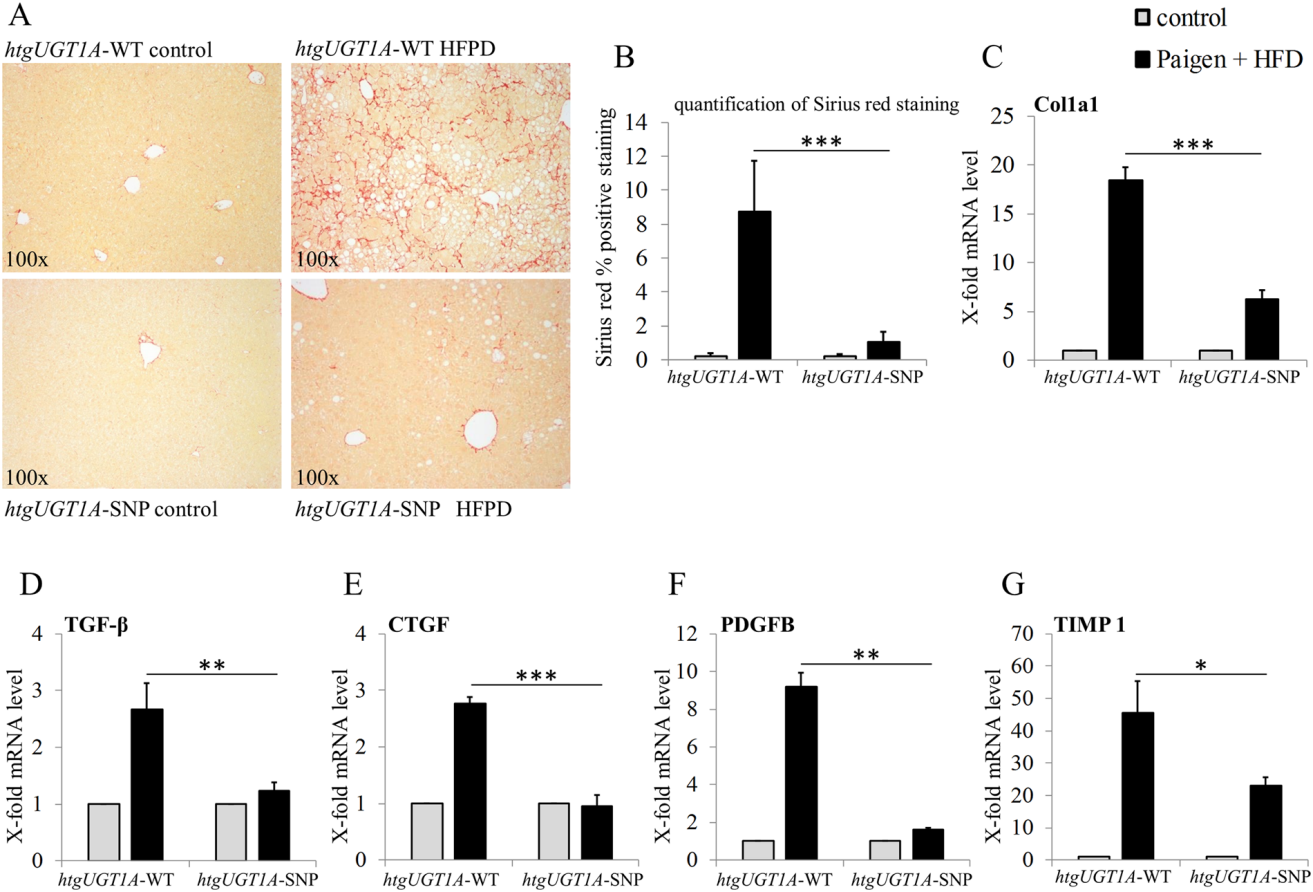
Assessment of liver fibrosis in *htgUGT1A*-WT and SNP mice after high-fat Paigen diet (HFPD) treatment. (**A**) Representative hepatic sections of histological Sirius red staining and (**B**) computational quantification of Sirius red stained areas. Hepatic collagen deposition was significantly lower in *htgUGT1A*-SNP mice. (**C–G**) Gene expression levels of the profibrotic biomarkers collagen type 1 alpha 1 (Col1a1), transforming growth factor beta (TGF-β), connective tissue growth factor (CTGF), platelet-derived growth factor subunit B (PDGFB) and tissue inhibitor metalloprotease 1 (TIMP 1) as indicators for severity of liver fibrosis. Transcriptional activation of all depicted fibrosis marker genes was significantly lower in *htgUGT1A*-SNP mice. In case of TGF-β and CTGF, minor transcriptional inductions were detected in HFPD-treated *htgUGT1A*-SNP mice. Graphs are expressed as means ± standard deviation. *p < 0.05; **p < 0.01; ***p < 0.001.

With the intention of determining differences in fibrosis development and hepatic collagen deposition, computational quantification of Sirius red staining and gene expression analysis of profibrotic biomarkers was evaluated. Histological staining showed a higher content of fibrillar collagens, and thus fibrosis development, in *htgUGT1A*-WT mice (Fig. 2A). Computational quantification of Sirius red staining further supported the histological finding and detected 8.4-fold higher percentage portion of red coloured fibrillar collagens in *htgUGT1A*-WT mice (Fig. 2B). Moreover, a 3.0-fold higher transcriptional activation of collagen type 1 alpha 1 (*Col1a1*) in *htgUGT1A*-WT mice supported the results at a molecular level and confirmed the differences in fibrosis development between both mouse lines (Fig. 2C).

Manifestation of liver fibrosis is usually associated with an increased expression of cytokines, chemokines and various key genes influencing fibrogenesis. In *htgUGT1A*-WT mice, HFPD administration caused significantly higher transcriptional activation of the profibrotic markers transforming growth factor beta (TGF-β), connective tissue growth factor (CTGF), platelet-derived growth factor subunit B (PDGFB) and tissue inhibitor metalloprotease 1 (TIMP1), whereas mRNA induction was reduced (TIMP1 and PDGFB) or absent (TGF-β and CTGF) in the presence of SNPs (Fig. 2D–G). In combination, these data suggest a protective effect of a common *UGT1A* SNP haplotype during diet-induced steatohepatitis, resulting in attenuated hepatic fibrosis and inflammation.

### Increased *UGT1A* expression in *htgUGT1A*-WT mice

**Figure 3:**
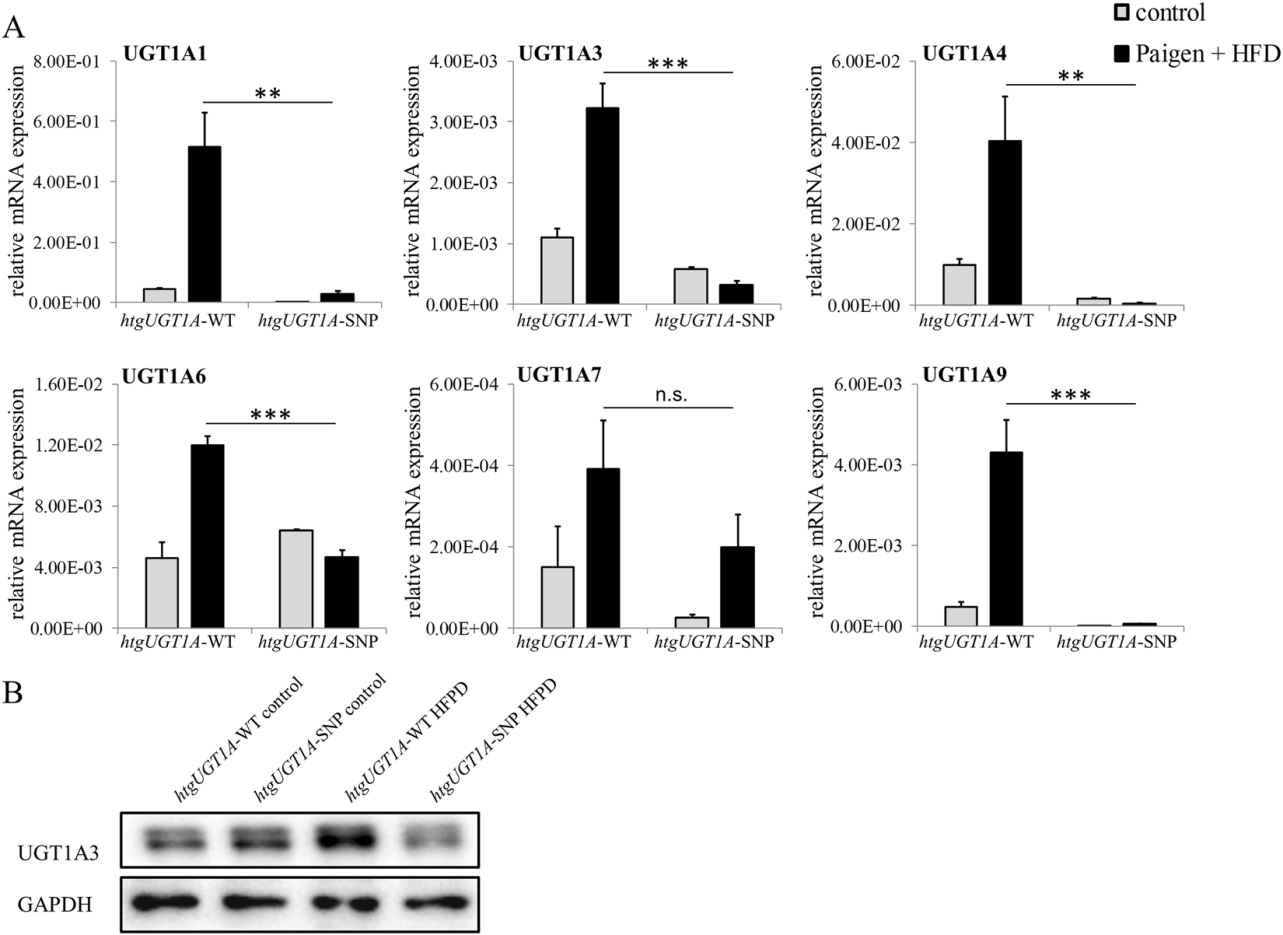
Hepatic *UGT1A* regulation in *htgUGT1A*-WT and SNP mice after high-fat Paigen diet (HFPD) treatment. (**A**) Hepatic mRNA expression levels of *UGT1A* isoforms relative to mouse β-actin. In *htgUGT1A*-WT mice, HFPD treatment led to a significant transcriptional upregulation of all depicted UGT1A isoforms, whereas in the presence of SNPs UGT1A induction was reduced (UGT1A1 and UGT1A9) or absent and remained below those observed in WT carriers. (**B**) Western blot analysis of hepatic UGT1A3 protein quantity. Higher protein amount was detected in *htgUGT1A*-WT mice. Graphs are expressed as means ± standard deviation. n.s. not significant; *p < 0.05; **p < 0.01; ***p < 0.001.

To further evaluate the potential mechanisms responsible for the observed protective effects in *htgUGT1A-*SNP mice, hepatic *UGT1A* expression was determined in both animal models (Fig. 3A). In the livers of *htgUGT1A*-WT mice, HFPD led to a significant upregulation of mRNA expression of all investigated *UGT1A* genes. In contrast and expectedly, significantly lower transcriptional activation was measured in *htgUGT1A*-SNP mice. Attention was given to the expression of *UGT1A3* representing the only UGT1A isoform capable of glucuronidating bile acids, which in turn are key regulators of nuclear receptors involved in glucose and lipid metabolism^26,27^. In line with the detected mRNA expression results, hepatic UGT1A3 protein quantity was markedly increased in HFPD treated *htgUGT1A*-WT mice (Fig. 3B).

### Reduced expression of FXR and PPARα in HFPD fed *htgUGT1A*-WT mice

**Figure 4:**
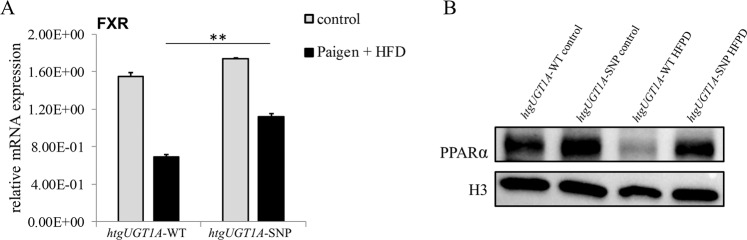
Hepatic mRNA expression and nuclear protein quantity of nuclear receptors in *htgUGT1A*-WT and SNP mice after high-fat Paigen diet (HFPD) treatment. (**A**) Enhanced inhibition of farnesoid X receptor (FXR) mRNA expression level was detected in *htgUGT1A*-WT mice (58%) compared to *htgUGT1A*-SNP mice (36%). (**B**) Western blot analysis of nuclear peroxisome proliferator-activated receptor alpha (PPARα) protein levels. Significantly reduced PPARα protein amount was measured in HFPD-treated *htgUGT1A*-WT, in contrast to unchanged levels in the presence of SNPs. Graphs are expressed as means ± standard deviation. *p < 0.05; **p < 0.01; ***p < 0.001.

FXR is known to play a crucial role in mediating effects of bile acids during NAFLD. After diet-induced liver injury, a significant decrease of the FXR mRNA expression was detected in both mouse lines (Fig. 4A). Interestingly, the degree of inhibition reached 58% in *htgUGT1A*-WT mice, compared to only 36% measured in *htgUGT1A*-SNP mice. PPARα is a downstream target of FXR and a well-known mediator of hepatic fatty acid oxidation^28^. The nuclear translocation of PPARα protein was downregulated in HFPD fed *htgUGT1A*-WT mice whereas PPARα activation remained unchanged in *htgUGT1A*-SNP mice (Fig. 4B). These results demonstrate a differential effect of *UGT1A* SNPs for the expression of nuclear receptors involved in cellular protection.

## Discussion

Effects of altered *UGT1A* expression on the pathogenesis of NAFLD and the associated consequences for the pathology of the liver have not been experimentally analysed. Contrary to our expectations and our original hypothesis, the results demonstrate that increased *UGT1A* expression does not protect against NASH progression in a humanized UGT1A animal model of NAFLD. The data suggest a protective effect of a common low-function *UGT1A* SNP haplotype in NAFLD/NASH. *HtgUGT1A*-SNP mice exhibited milder hepatic steatosis, significantly lower levels of hepatic triglycerides and a less pronounced elevation of AST and ALT levels compared to *htgUGT1A*-WT mice. Furthermore, a reduced deposition of fibrillar collagens and decreased expression levels of profibrotic and proinflammatory marker genes underscore an attenuated process of liver fibrosis in *htgUGT1A*-SNP mice. This was accompanied by the significant upregulation of *UGT1A* expression levels in *htgUGT1A*-WT mice, compared to a reduced or absent induction in mice carrying the low-function SNP variant.

To date, contradictory results have been published in various cohort-studies investigating an association between genetic *UGT1A* variants and human NAFLD. In line with our study, a decreased risk of paediatric NAFLD has been reported in 234 obese Taiwanese children associated with a low-activity *UGT1A1* gene variant^29^. Similarly, a case-control study with 641 adult patients suspected to have NAFLD reported an inverse association between unconjugated hyperbilirubinemia and the histopathological severity of liver damage in NASH^30^. In contrast, genome-wide association studies and other genetic studies of human NAFLD have failed to find an association between *UGT1A1* polymorphisms and NAFLD^31^. A likely explanation for the inconsistent data reported in these studies may involve the presence of *UGT1A* polymorphisms found in other isoforms than UGT1A1. Since many SNPs exist in linkage-disequilibrium with each other, the inter-individual *UGT1A* sequence variation is highly variable^22^. Previously, we were able to show that 76% of homozygous UGT1A1*28 carriers, a genotype that is associated with elevated bilirubin levels, were simultaneously homozygous for 9 other *UGT1A* polymorphisms^32^. Therefore, a combination of multiple polymorphisms in distinct UGT1A isoforms is suggested to be responsible for the observed hepatoprotective effects in this study and maybe also for the differential results in human population studies only considering polymorphisms in the *UGT1A1* gene.

A molecular mechanism potentially triggering the *UGT1A* SNP-associated protective effects could include the impaired glucuronidation of molecules that function as ligands for nuclear receptors (i.e. FXR or PPARα) involved in lipid homeostasis. A specific example is the UGT1A-mediated glucuronidation of dietary fatty acids, such as arachidonic acids and its metabolites 20-hydroxyeicosatetraenoic acid (20-HETE) and leukotriene B4 (LTB4), which have been previously identified as potent PPARα activators^33^ and as UGT1A3 or UGT1A9 substrates^14,34,35^. In this context, *Little et al*. suggested that UGT1A enzymes may function as modulators for the availability of fatty acids to act as ligands for nuclear receptors. Therefore, the decreased *UGT1A* expression in many different isoforms, as present in *htgUGT1A*-SNP mice, may lead to higher intracellular levels of ligands involved in a broad array of lipid homeostasis signalling pathways. The impaired capacity of *htgUGT1A*-SNP mice to eliminate potential FXR or PPARα ligands may improve the PPARα-mediated mitochondrial fatty acid oxidation, hepatic fatty acid uptake, peroxisomal beta-oxidation or other crucial processes of lipid metabolism and leads to the lower degree of hepatic steatosis. Moreover, polymorphisms in the specific UGT1A3 isoform, which is the major enzyme in the *UGT1A* gene locus capable to catalyse the glucuronidation of bile acids, are likely to also contribute to the beneficial effects and mediated independently of the UGT1A1*28 promoter polymorphism. Bile acid levels were shown to be elevated in patients with NASH^36^ and have been identified as FXR inducers^37^. Due to higher *UGT1A3* expression in *htgUGT1A*-WT animals potentially leading to increased glucuronidation of FXR-inducing bile acids, unaffected UGT1A3 activity is suggested to reduce FXR activation leading to an impaired FXR-induced PPARα-mediated fatty acid oxidation. Reduced FXR expression as well as lower levels of nuclear PPARα protein in *htgUGT1A*-WT mice underline this hypothesis and lead to the conclusion that the differential regulation of both nuclear receptors is likely to be directly linked to the differential expression of *UGT1A* genes. Although glucuronidation of bile acids has previously been demonstrated in mice^38^, it is important to note that bile acid glucuronidation only constitutes a relevant detoxification pathway in humans. In mice, however, it is predominantly converted into muricholic acid and only minute amounts are present^39,40^. Even though the relative occurrence of this conjugation reaction is minor in mice, the proposed physiological role is likely to occur in humans. In addition, the FXR downstream targets small heterodimer partner and PPARα have previously been shown to initiate the downregulation of the lipogenic master regulator sterol regulatory element-binding protein 1c (SREBP-1c)^28,41^. Therefore, increased FXR expression in *htgUGT1A*-SNP mice might be associated with reduced SREBP-1c activation and consequently with a downregulation of *de novo* lipogenesis.

As a consequence, the combination of two or more polymorphisms in different UGT1A isoforms may be a more accurate indicator for the occurrence of *UGT1A* SNP-associated liver protection during NAFLD or NASH in human population studies. Along with PPARα and FXR, other nuclear receptors such as constitutive androstane receptor, pregnane X receptor, liver X receptor and hepatocyte nuclear factor 4 have also been implicated in the pathogenesis of NAFLD^24^ while also being involved in the transcriptional regulation of *UGT1A* genes^42^. Therefore, polymorphisms in *UGT1A* genes may ultimately lead to various metabolic changes in patients with NAFLD and large-scale OMICS analysis might be necessary to get a full picture of the transcriptomic and proteomic alterations associated with this common *UGT1A*-SNP genotype. However, we cannot fully exclude that the observed effects are influenced by the murine Ugt1a enzymes which are also expressed in *htgUGT1A*-WT and SNP mice. This leads to an increased glucuronidation capacity in both mouse lines, which is lower in *htgUGT1A-*SNP mice. Therefore, further studies with *Ugt1a* knockout mice simultaneously carrying the human *UGT1A*-WT or *UGT1A-*SNP transgene would be needed to exclude the effects of non-physiologically high levels of glucuronidation.

In conclusion, our data indicate a protective effect of a Gilbert syndrome-associated *UGT1A* haplotype leading to milder hepatic steatosis during the development of NASH. Higher expression of UGT1A enzymes was observed in *htgUGT1A-*WT mice, while *htgUGT1A*-SNP mice showed lower serum aminotransferase levels and reduced hepatic collagen deposition. Due to the decreased PPARα protein and lower FXR expression levels in *htgUGT1A*-WT mice, we hypothesize that increased *UGT1A* expression may lead to the facilitated elimination of potential ligands involved in lipid homeostasis.

## Methods

### Animal model and experimental design

For animal experiments, previously described 8–10 week-old *htgUGT1A*-WT and SNP mice were used^32,43^. As both mouse lines contain the human *UGT1A* transgene in addition to the murine *Ugt1a* gene locus, the glucuronidation capacity of both animal models is likely above the physiological levels in non-transgenic C57Bl/6 mice, which is, due to the presence of 10 common *UGT1A* polymorphisms, significantly lower in *htgUGT1A*-SNP mice. Male mice of each genotype were divided into two groups consisting of six to eight animals. Both groups were either fed a regular chow or a HFPD for 24 weeks. The HFPD was purchased from the Altromin GmbH & Co. KG Company (Seelenkamp, Germany) and contained a raw fat content of 42% in which fat contributes 70% of total energy requirements. This diet is further characterized by the addition of 1.25% cholesterol and 0.5% sodium cholate. All mice had *ad libitum* access to water and chow and were kept at 22 °C with a 12 hour day/night cycle in the Central Animal Facility of the University Hospital Bonn. 10 days before HFPD administration *htgUGT1A* mice received a 4% raw fat containing diet to adapt animals to the change of dietary components. All experiments were performed and in accordance to the “German Animal-Protection Law” and the relevant guidelines of the Local Institutional Animal Care unit of our university (Haus für experimentelle Therapie, Bonn, Germany) and approved by the relevant North Rhine-Westphalian state-agency for Nature, Environment and Consumer Protection (LANUV, Germany) under the file reference LANUV 84-02.04.2016.A483.

### Tissue collection and biochemical analysis

After HFPD treatment, animals were sacrificed for organ and blood collection. For biochemical analysis of serum ALT and AST activities, the collected blood was centrifuged at 4.800 rpm for 10 min to remove blood cells. The supernatant was stored at −20 °C until analyzation by means of a Fuji DRI-CHEM NX500i serum analyser.

Organ samples were immediately snap-frozen in liquid nitrogen and stored at −80 °C until use. The right lateral lobe was separated before, fixed in 4% paraformaldehyde for three days, subsequently embedded in paraffin and then used for pathohistological examinations.

### Histological staining and computational analysis

Paraffin-embedded liver sections were trimmed into 2 µm thick slices, and stained in 0.1% Sirius red solution (DirectRed 80 in saturated picric acid) for detection of fibrous collagen tissue. The Sirius red positive stained area was quantified using ImageJ software (U.S. National Institutes of Health; http://rsb.info.nih.gov/ij/) and shown as percentage positive staining of the total section area. Images were analysed from four randomly selected images (magnification 100×) of each animal and were averaged. H&E staining was applied to visualize lipid-droplets within hepatocytes, cellular ballooning and infiltration of inflammatory cells according to a standard protocol procedure with minor modifications^44^.

### Triglyceride measurement

Hepatic triglyceride levels were photometrically determined using the Triglyceride Colorimetric Assay Kit (Cayman Chemicals). 300 mg snap-frozen liver tissue was hydrolysed according to manufacturer’s instructions and analysed in duplicates using a MultiSkan GO microplate reader at 540 nm.

### Gene expression analysis

RNA isolation from snap-frozen liver samples, cDNA synthesis and gene expression analysis by qPCR were performed as described previously^43^. The assays listed below were purchased from Thermo Scientific and used for quantification of inflammatory and fibrosis-related gene expression levels. These include C-C chemokine ligand 2 (CCL2; Mm00441442_m1), tumour necrosis factor alpha (TNF-α; Mm00443260_g1), collagen type 1 alpha 1 (Col1a1; Mm00801666_g1), transforming growth factor beta (TGF-β; Mm01178820_m1), platelet-derived growth factor subunit B (PDGFB; Mm00440677_m1) connective tissue growth factor (CTGF; Mm01192933_g1) and tissue inhibitor metalloprotease 1 (TIMP1; Mm01341361_m1). Expression levels were normalized relative to mouse beta-actin and expressed as fold mRNA levels compared to untreated *htgUGT1A*-WT or SNP mice.

### Western blot analysis

60 mg of frozen liver tissue was homogenized in RIPA extraction buffer containing protease inhibitor cocktail and subsequently incubated for 1 h on a shaking plate at 4 °C. After centrifugation (13.000 rpm, 10 min, 4 °C), the supernatant was used for Western blot analysis. Nuclear extraction tissue specimens were prepared using Nuclear Extraction Kit (Abcam) according to manufacturer’s instructions. For SDS-PAGE separation, 30 µg of protein was boiled at 95 °C for 5 min in Laemmli sample buffer, separated on a 10% acrylamide gel and blotted onto a nitrocellulose membrane *via* electrotransfer using the Trans-Blot®-Turbo transfer system. Incubation with primary antibodies (anti UGT1A3, Abnova H00054659-M02, anti GAPDH, Santa Cruz sc-32233, anti PPARα, Santa Cruz sc-398394 and anti H3 Abcam (ab12079)) was carried out in 5% dry milk. Appropriate secondary antibodies (Santa Cruz, sc-516102 and sc-2054) were used and protein was visualized by chemoluminescence with the use of ChemiDOC MP imaging system (Supplementary Figure S1,S2).

### Statistical analysis

Data are expressed as mean ± SD determined by one-way analysis of variance followed by Students *t*-test to define significance. A pool of six to eight mice in each HFPD group was analysed (four mice per control group); p values below 0.05 were considered as statistically significant.

## Supplementary information

Supplementary Information.
