# Supplementary material for: A Gilbert syndrome-associated haplotype protects against fatty liver disease in humanized transgenic mice
Source: Sci Rep. 2020 May 26;10:8689. doi: 10.1038/s41598-020-65481-4 (PMC7250928; doi:10.1038/s41598-020-65481-4)
Supplement: Supplementary file 1 — Supplementary Information. [file 41598_2020_65481_MOESM1_ESM.pdf]

A Gilbert syndrome-associated haplotype protects against fatty liver disease in humanized transgenic mice

Steffen Landerer, Sandra Kalthoff, Stefan Paulusch, Christian P. Strassburg*

Department of Internal Medicine I, University Hospital Bonn, 53127 Bonn, Germany

* **Corresponding authors**

Correspondence: Christian P. Strassburg, MD

Department of Internal Medicine I,

University Hospital Bonn,

Venusberg-Campus 1,

53127 Bonn, Germany

Telephone: +49 22828715216, Fax: +49 22828719657

Email: christian.strassburg@ukbonn.de

Contributing authors:

Steffen Landerer: steffen.landerer@ukbonn.de

Sandra Kalthoff: sandra.kalthoff@ukbonn.de

Stefan Paulusch: stefan.paulusch@ukbonn.de

Supplementary figure S1

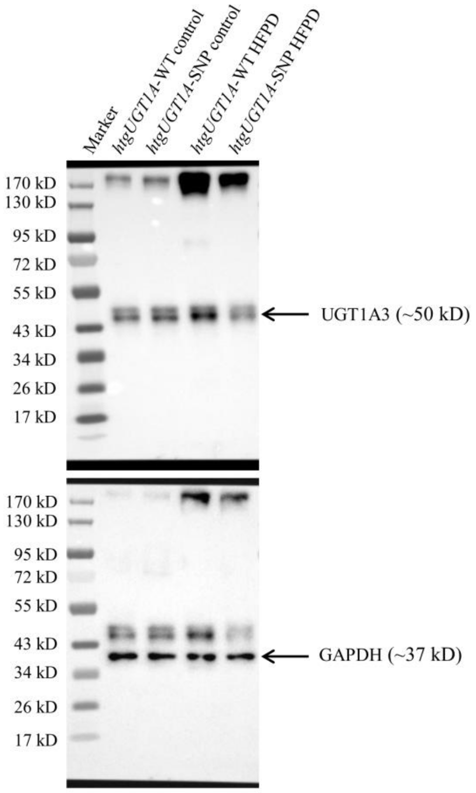

Supplementary figure S2

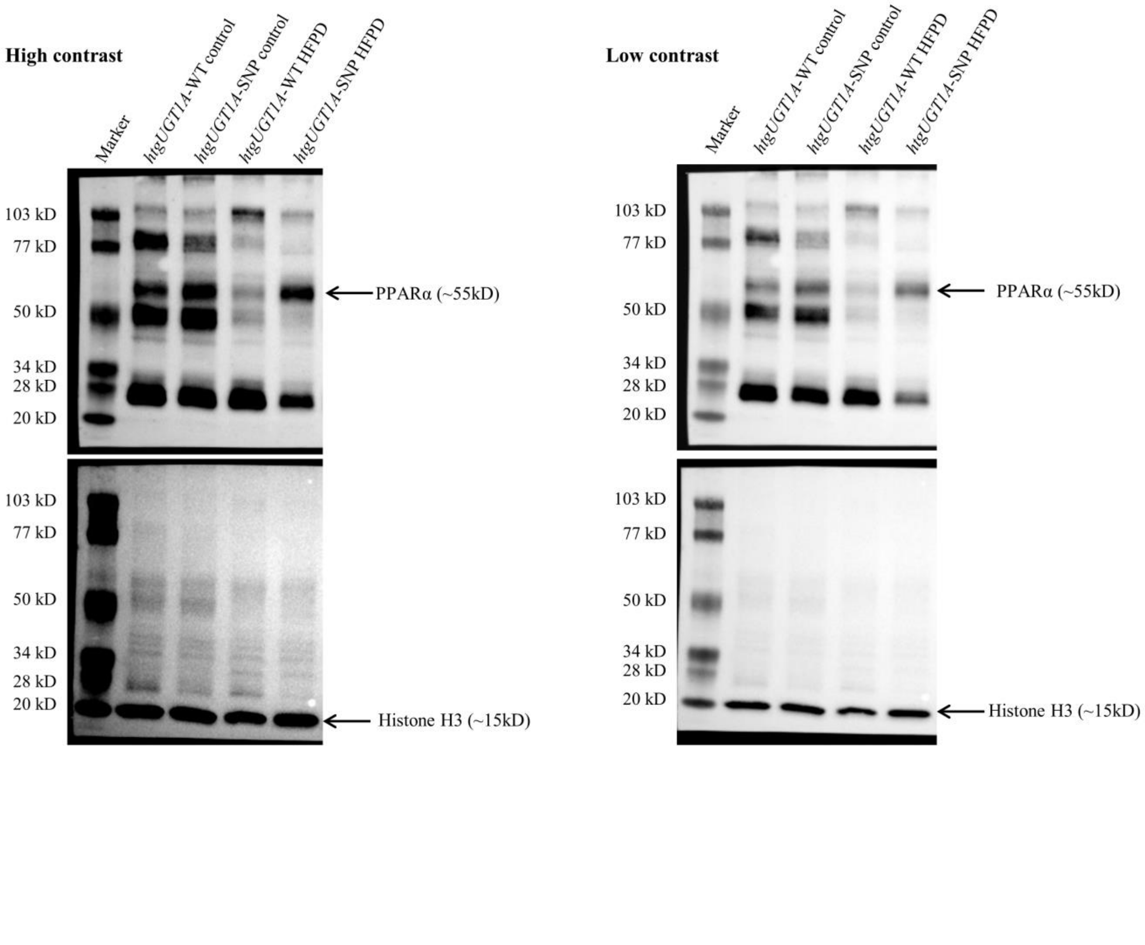

Figure Legends Supplementary figures

Fig. S1: Western blot analysis of hepatic UGT1A3 protein quantity. Higher protein amount was detected in *htgUGT1A*-WT mice.

Fig. S2: Western blot analysis of nuclear peroxisome proliferator-activated receptor alpha (PPARα) protein levels (high contrast left panel, low contrast right panel).
